# Associations Between Self-Compassion and Behavioural Intention to Receive Seasonal Influenza, Pneumococcal and Respiratory Syncytial Virus Vaccination Among Community-Living Older Adults in Western China: A Population-Based Cross-Sectional Survey

**DOI:** 10.3390/vaccines14060513

**Published:** 2026-06-06

**Authors:** Hongbiao Chen, Sinawaer Abulimiti, Miaoqi Wan, Fuk-yuen Yu, Yuan Fang, He Cao, Fengjuan Chen, Jimileguli Aini, Xiaoqian Deng, Haiyan Yan, Aynur Yusup, Abuduwupur Kahar, Zixin Wang

**Affiliations:** 1 Longhua District Centre for Disease Control and Prevention (Longhua District Health Supervision Institute), Shenzhen 518055, China; gesila2021@163.com (H.C.); 13428999260@163.com (H.C.); 2 Kashgar Municipal Center for Disease Control and Prevention (Health Inspection Institute), Kashgar 844000, China; 760582691@139.com (S.A.); 17399331255@163.com (J.A.); 18623028860@163.com (X.D.); 15739117321@139.com (H.Y.); 18109989355@163.com (A.Y.); 13579091300@163.com (A.K.); 3 Centre for Health Behaviours Research, JC School of Public Health and Primary Care, The Chinese University of Hong Kong, Hong Kong, China; 1155173623@link.cuhk.edu.hk (M.W.); benfyyu@cuhk.edu.hk (F.-y.Y.); lunajoef@gmail.com (Y.F.); 4 Dapeng District Centre for Disease Control and Prevention, Shenzhen 518119, China; cathychan99@163.com

**Keywords:** seasonal influenza vaccination, pneumococcal vaccination, respiratory syncytial virus vaccination, self-compassion, older adults, Western China

## Abstract

**Background:** Self-compassion is the practice of treating oneself with kindness and understanding during times of hardship. There is a lack of studies investigating the associations between self-compassion and vaccination behaviors. This study investigated the associations between self-compassion and behavioral intention to receive seasonal influenza vaccination (SIV), pneumococcal vaccination (PV), and respiratory syncytial virus (RSV) vaccination among community-living older adults in Western China. **Methods:** A cross-sectional survey was conducted among people aged ≥60 years in Kashgar, China between January and February 2026. Participants were recruited through multi-stage random sampling. Multivariable logistic regression models were fitted. **Results:** Among all participants, 56.5% intended to receive a fully subsidized SIV in the next year. Among those without a prior vaccination history, 48.2% and 49.7% intended to receive fully subsidized PV and RSV vaccination in the next year, respectively. After adjusting for significant background characteristics, higher levels of self-compassion (e.g., higher levels of mindfulness, self-kindness and common humanity, and lower levels of over-identification, isolation and self-judgment) were associated with higher odds of behavioral intention to receive a fully subsidized SIV, PV and/or RSV vaccination. **Conclusions:** Our findings suggested a new angle to promote vaccination uptake. Future studies may evaluate the efficacy of self-compassion interventions in improving vaccination uptake among older adults.

## 1. Introduction

Seasonal influenza infection, pneumococcal diseases, and respiratory syncytial virus (RSV) infection are health threats for people aged 60 years or above. Worldwide, seasonal influenza is one of the most common respiratory infections [1,2]. The risk of severe outcomes of seasonal influenza increases markedly with age. In China, an estimated 80% of influenza-associated excess respiratory deaths occur in adults aged 60 years or above, with a mortality rate of 38.5 per 100,000 per season [3]. Xinjiang Uygur Autonomous Region, located in Western China, has a high incidence of seasonal influenza infection due to its cold and low-humidity climate, and the surveillance data has shown a rapid increase in seasonal influenza cases in this region in recent years [4]. *Streptococcus pneumoniae* is the most common cause of pneumococcal diseases (PDs) and invasive PD (IPD) [5]. Individuals aged 60 years or above are at much higher risk of PD and IPD and have the highest risk of death caused by IPD [6]. Moreover, secondary and co-infection of bacterial pneumonia (mostly caused by *Streptococcus pneumoniae*) following seasonal influenza is common among older adults and is associated with higher mortality and morbidity [7]. Approximately 49% of deaths caused by PD or IPD in China were concentrated in Western China [8]. This region keeps reporting the highest mortality rate of PD in China [8]. Respiratory syncytial virus infection causes infection of the airway, lungs, and middle ear [9]. Adults aged 60 years or above are more susceptible to severe consequences caused by RSV infection, including bronchiolitis, pneumonia, and respiratory failure [10]. Older adults with chronic medical conditions have a much higher risk of RSV-associated hospitalization and mortality [10]. About 16.8% of medically attended acute respiratory infections in mainland China are attributed to RSV infection [11]. RSV infection has shown a significant resurgence, with an over 300% increase in respiratory pathogen positivity reported in Western China by 2024 [12].

Vaccination is an effective strategy for preventing infection and reducing the mortality rate after infection. Seasonal influenza vaccination (SIV) and pneumococcal vaccination (PV) could effectively prevent influenza and its complications, and vaccine-type PD and IPD among older adults without safety concerns [13,14]. The World Health Organization (WHO) and health authorities in China recommend that adults aged 60 years or above receive an SIV every year and receive a PV in their lifetime [15,16]. However, the coverage of SIV and PV remained inadequate among older adults in China. The overall SIV coverage among adults aged 60 years or above in China was 30.0% in the 2023/24 season and 34.0% in the 2024/25 season [17,18], which was much lower than the 75% coverage target set by the WHO [19]. A meta-analysis indicated that the overall PV coverage was 21.7% in mainland China and only 5.5% among adults aged 60 years or above [20]. Regional differences in PV coverage were also observed, with lower coverage in Western China than in Central or Eastern regions [20]. Therefore, health promotion to increase SIV and PV among older adults, especially those living in Western China, is needed.

Although hospitalization and mortality can come after RSV infection [10], vaccines have only been available in recent years. Two RSV vaccines, the AREXVY (GSK, Durham, NC, USA) and the ABRYSVO^TM^ (Pfizer Inc., New York, NY, USA), were both effective in preventing RSV-related emergency department visits and hospitalizations in adults aged 60 or above without safety concerns [21]. RSV vaccines were approved for use in older adults aged 60 years or above in Macau and Hong Kong in 2024 [22]. However, such vaccines were not available in mainland China (including Xinjiang) at the time when this study was conducted. Several ongoing clinical trials are evaluating the effectiveness and safety of RSV vaccines in mainland China [23]. It is expected that the RSV vaccines will become available in the country in 2026.

For promoting vaccination uptake, identifying related determinants is crucial for calibrating promotion strategies. Numerous studies were conducted to investigate determinants of SIV or PV uptake among older adults in China [17,18,23,24,25,26,27,28,29,30,31]. These studies focused on modifiable determinants concerning knowledge related to seasonal influenza or pneumococcal diseases, perceived risk and severity of these diseases, attitudes related to SIV or PV (e.g., perceived benefits, concerns related to side effects and inconvenience, recommendation of family and doctors, perceived self-efficacy), and trust in service providers. However, most of these studies were conducted in Eastern or Southern China, and there is a lack of studies investigating determinants of SIV or PV uptake among older adults in Western China, including Xinjiang. To our knowledge, only two published studies investigated factors associated with behavioral intention to receive an RSV vaccine among older adults in China [23,32]. Modifiable determinants identified by these studies included attitudes toward RSV and the vaccine (perceived risk and severity of RSV, perceived vaccine efficacy, concern about side effects, recommendations from significant others, and perceived self-efficacy) and altruistic factors (general altruism and family member-oriented altruism) [23,32]. However, neither of these two studies involved older adults in Xinjiang.

Beyond the aforementioned modifiable determinants, a new candidate, namely self-compassion, may be worthy of further investigation and shed light on a new framework for health promotion. Self-compassion is the practice of treating oneself with kindness and understanding during times of hardship, whether the source is internal failure or external circumstances [33]. Self-compassion is defined as a multifaceted system with three interconnected domains: emotionally responding to pain and failure (self-kindness versus self-judgment), cognitively understanding one’s predicament (as common humanity versus isolation), and paying attention to suffering (mindfulness versus over-identification) [34]. Self-compassion has been proven to be a powerful tool for bouncing back from health challenges and, for those with chronic health conditions, developing better adaptive coping styles [33]. Previous studies indicated that a higher level of self-compassion was associated with a higher likelihood of performing healthy behaviors, including quitting smoking, eating a healthy diet, exercising, and seeking medical care, and thus reduced the risk of disease and mortality [35]. Individuals with a higher level of self-compassion tend to engage in a wide range of healthy behaviors that subsequently improve physical health [36]. One study reported that individuals who have received a COVID-19 vaccination had a significantly higher level of self-kindness, common humanity, and mindfulness, as compared to unvaccinated people [37]. Accordingly, it is possible that a higher level of self-compassion is associated with higher motivation to receive vaccination. However, such a hypothesis was not tested in previous studies.

**Figure 1:**
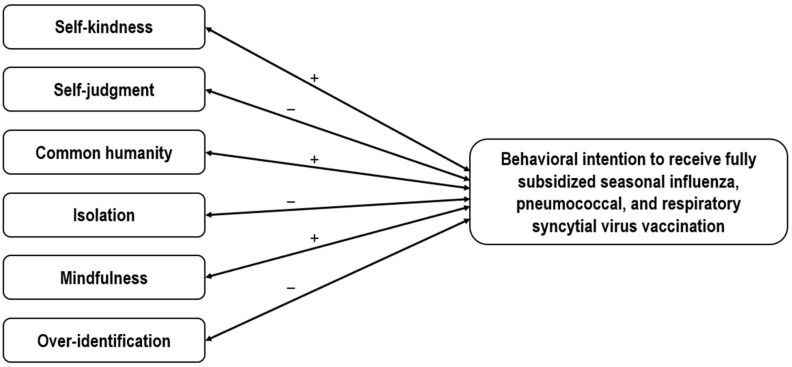
Conceptual diagram of the relationships between different domains of self-compassion and vaccination intention.

To address the knowledge gaps, the present study examined the associations between the level of self-compassion and behavioral intention to receive fully subsidized SIV, PV, and RSV vaccines among a random sample of older adults aged 60 years or above in Western China. We hypothesized that higher levels of self-compassion would be associated with higher odds of behavioral intention to receive these three types of vaccination. A conceptual diagram is presented in Figure 1.

## 2. Methods

### 2.1. Study Design

A cross-sectional survey among people aged 60 years or above was conducted in Kashgar, China, between January and February 2026. Kashgar is a city in the Tarim Basin region of southern Xinjiang. It is one of the westernmost cities in mainland China, bordering Kyrgyzstan and Tajikistan. The city has a population of 1.01 million in 2025; over 92% of its residents are Uyghur. At the time when this study was conducted, self-financed SIV (CNY100 or US$14.5 per dose) and PV (CNY600 or US$87 per dose) were provided to Kashgar residents aged ≥60 years. Recently, many provinces in China started to provide free SIV and PV to older adults [38]. It is expected that Xinjiang will introduce a new policy by providing free SIV and PV to older adults in the near future. RSV vaccines are not yet available in mainland China. It is expected that the RSV vaccines will become available in the country in 2026.

### 2.2. Participants and Recruitment

The inclusion criteria were: (1) aged 60 years or above, (2) living in Kashgar at the time of the survey, and (3) able to communicate in Mandarin Chinese or Uyghur. In Kashgar, health service centers (23 in total) provide comprehensive health-related services to local residents. These centers keep service records of all residents living in the areas including basic sociodemographic information, contact information, chronic disease diagnosis and management, and information related to pregnancy and childbirth (for women only). These health service centers utilize “Family Doctor Contract Services”, and dedicated service providers are assigned to follow up and manage the health of each family. These service providers proactively reach out to local families on a regular basis. Therefore, the staff of the health service centers have established rapport with local residents, which may increase the response rate of this study.

In this study, all 23 health service centers facilitated the subject recruitment. Simple random sampling was used to recruit participants. Each health service center randomly selected 35 residents aged 60 years or above from its service records and then called the residents at different timeslots during weekdays and weekends. Prospective participants were briefed about the study and invited to visit the center for a face-to-face interview. Prior to the start of the interview, staff of the health service center obtained participants’ written informed consent and performed a 30 min interview in Mandarin Chinese or Uyghur. After completion of the interview, each participant would receive a cash coupon of CNY25 (US$3.5) as a token of appreciation. Ethics approval was obtained from the Chinese University of Hong Kong (reference: SBRE-25-0487).

### 2.3. Sample Size Planning

The target sample size was 600. We followed the method that was commonly used in published studies to estimate the sample size [32]. We assumed that 10–50% of participants without a facilitating condition (the reference group) would have an intention to receive vaccination. The sample size allowed us to detect a smallest odds ratio of 1.5 between participants with and without a facilitating condition (power of 0.80 and an alpha of 0.05, PASS 11.0, NCSS, LLC). We conservatively assumed that 75% of the eligible people being invited would participate in this study. Therefore, we needed to invite 800 older adults (about 35 in each health service center) to join the study.

### 2.4. Measurements

#### 2.4.1. Development of the Questionnaire

A panel consisting of experts in public health, vaccination behaviors and health psychology, staff of the Center for Disease Control and Prevention and Health Service Centers, and older adults was formed to develop the questionnaire. The questionnaire was in simplified Chinese. We conducted face-to-face interviews of 10 residents aged 60–80 years to test the length of the questionnaire and the logistics of data collection. According to the participants in the pilot study, the questions were easy to understand, and the duration of the interview (around 20–35 min) was acceptable. Based on the minor suggestions made by the participants, the panel finalized the questionnaire.

#### 2.4.2. Background Characteristics

Participants reported sociodemographic characteristics, including age, sex assigned at birth, education level, marital status, employment status, and living arrangement. In addition, information on lifestyles (smoking and binge drinking in the past year), presence of chronic conditions, and history of confirmed seasonal influenza, pneumonia, RSV, and SARS-CoV-2 infection was collected. In addition, information on uptake of SIV in the previous flu season (since September 2025) and history of lifetime PV, RSV and COVID-19 vaccination were collected.

#### 2.4.3. Behavioral Intention to Receive Different Types of Vaccination

Participants were asked about their likelihood of receiving a fully subsidized SIV provided by the government in the next year. Participants without or uncertain about a prior history of PV were asked about their likelihood of receiving a fully subsidized PV provided by the government in the next year. Regarding RSV vaccination, participants without or uncertain about a prior history of RSV vaccines were briefed about RSV vaccines before measuring their likelihood of receiving a fully subsidized RSV vaccination provided by the government in the next year. The contents of the briefing were “Receiving RSV vaccination could reduce the risk of RSV infection by 80% among people aged 60 or above. In older adults with chronic diseases, RSV vaccination could reduce RSV infection by 95%. Older adults only need to receive one dose of the RSV vaccination, and the duration of protection is at least two years. RSV vaccination is not yet available for older adults in China”. Similar briefing contents were used in a published study [32]. Measuring behavioral intention under the condition that participants knew the efficacy and availability of the vaccine could facilitate a better interpretation of results. Pre-survey briefing was widely used in surveys investigating attitudes or intentions to receive vaccination [39]. The response categories of the aforementioned questions were 1 = very unlikely, 2 = unlikely, 3 = neutral, 4 = likely, and 5 = very likely. Participants who answered “likely” or “very likely” were defined as having a behavioral intention to receive vaccination. Such a definition was commonly used in published studies [32,39].

#### 2.4.4. Self-Compassion

Level of self-compassion was assessed using the validated Chinese version of the Self-Compassion Scale–Short Form (SCS-SF) [40]. The SCS-SF is a self-reported measure that has 12 items and measures six components of self-compassion (2 items per component). These components are self-kindness (care about oneself with warmth), common humanity (all humans face challenges and experience suffering), mindfulness (neither exaggerating nor understating negative experience), self-judgment (criticizing oneself), isolation (thinking suffering only happens to oneself but not others), and over-identification (rumination involving exaggeration of negative experience). Each item is rated by a 5-point Likert scale (from 1 = almost never to 5 = almost always). The score of each component was calculated by summing up two individual item scores. Items of negative components (self-judgment, isolation, over-identification) were reverse-scored when calculating the total score of each component. The possible range of scores for each component was between 2 and 10. Higher scores in self-kindness, common humanity, and mindfulness represented higher levels of self-kindness, common humanity, and mindfulness. Higher scores for self-judgment, isolation and over-identification indicated a lower level of self-judgment, isolation and over-identification.

### 2.5. Statistical Analysis

We calculated the mean and standard deviation (SD) of the scale or item scores. Behavioral intention to receive fully subsidized SIV, PV and RSV were used as dependent variables. Univariate logistic regression models were used to investigate the associations between background characteristics and each of the dependent variables. After adjusting for significant background characteristics, associations between different components of self-compassion and the dependent variables were examined using multivariable logistic regression models. Crude odds ratios (ORs), adjusted odds ratios (AORs) and their 95% confidence interval (CI) were obtained; a *p*-value less than 0.05 was considered statistically significant. Hosmer and Lemeshow goodness-of-fit tests were conducted to evaluate the strengths of the logistic regression models in this study. The insignificant test results (*p* > 0.05) indicated that the model’s estimates fit the data at an acceptable level. SPSS version 26.0 (IBM, Armonk, NY, United States) was used for data analysis.

## 3. Results

### 3.1. Background Characteristics of the Participants

Among 805 eligible adults aged ≥60 years approached by the research team, 113 declined to participate because of time constraints or other practical reasons, and 692 completed the face-to-face interview. The response rate was 86.0% (692/805). The majority of the participants were 60–70 years old (68.6%), female (51.9%), of Uyghur ethnicity (92.5%), married (78.3%), and retired (76.2%) and had an educational level of junior high or below (85.5%) and a monthly personal income below 3000 CNY (US$426.1) (70.4%). About half of them were living with their spouse (60.3%) and children (42.2%). Among the participants, 16.5% and 5.2% reported smoking and binge drinking in the past year, respectively. Over 70% had at least one chronic condition. The most prevalent chronic condition was hypertension (61.0%), followed by other cardiovascular diseases (22.8%) and diabetes mellitus (18.9%). Over half of them reported a history of confirmed SARS-CoV-2 infection (63.9%), 14.2% reported confirmed seasonal influenza infection in the 2025-26 flu season (since September 2025), and 2.7% and 6.2% had confirmed pneumococcal diseases and RSV infection in their lifetime. During the time of the current study, 12% had received SIV since September 2025 and 15.2% had taken up a PV in their lifetime. None of the participants received an RSV vaccine (Table 1).

### 3.2. Descriptive Statistics of Dependent Variables and Independent Variables of Interest

Among all participants, 56.5% intended to receive a fully subsidized SIV provided by the government in the next year. Among 587 participants without a prior history of receiving PV, 48.2% intended to receive a fully subsidized PV provided by the government in the next year. After being briefed about the facts of the vaccine, 49.7% intended to receive a fully subsidized RSV vaccine provided by the government in the next year. The mean and standard deviation (SD) of different domains of self-compassion are presented in Table 2.

### 3.3. Factors Associated with Behavioral Intention to Receive Fully Subsidized SIV, PV and RSV Vaccination

Living with a spouse (OR: 1.48–2.02, *p* values: <0.001 to 0.02) and uptake of the COVID-19 vaccination primary series (OR: 2.18–3.09, *p* values: <0.001 to 0.001, reference group: 0–1 dose) or booster doses (OR: 1.88–3.16, *p* values: <0.001 to 0.003) were associated with higher odds of intending to receive fully subsidized SIV, PV and RSV vaccinations. Those who received SIV since September 2025 had a higher intention to receive SIV and PV in the next year (OR: 1.81 and 4.01, *p* = 0.02 and <0.001), while being employed was associated with a lower intention to receive SIV and RSV vaccines (OR: 0.47 and 0.64, *p* values: <0.001 and 0.01). Participants with Han ethnicity (OR: 2.83, *p* = 0.003, reference group: Uyghur ethnicity) and those who had higher education (college or above: OR: 2.30, *p* = 0.04, reference group: junior high or below) were more likely to report a behavioral intention to receive SIV. Those who were living with children (OR: 1.68, *p* < 0.001) or grandchildren (OR: 1.76, *p* = 0.002) and with a history of any chronic condition (OR: 1.71, *p* = 0.002) had higher odds of intending to receive an RSV vaccine. However, a negative correlation was observed between history of confirmed SARS-CoV-2 infection and intention to receive an RSV vaccine (OR: 0.69, *p* = 0.02). In addition, a history of PD was associated with lower odds of intending to receive PV (OR: 0.19, *p* = 0.03) (Table 3 and Appendix A).

After adjusting for significant background characteristics, higher levels of mindfulness (*p* values: <0.001 to 0.004) and lower levels of over-identification (*p* values: <0.001 to 0.01) were associated with higher odds of intending to receive an SIV, PV and RSV vaccine. Participants who reported a higher level of self-kindness (both *p* < 0.001) and a lower level of isolation (*p* = 0.001 and <0.001) had a higher intention to receive an SIV and PV. Those who had a lower level of self-judgment had a higher likelihood of intending to receive an SIV and RSV vaccine (*p* = 0.01 and 0.002). In addition, a positive correlation between common humanity items (higher level of common humanity) and behavioral intention to receive an SIV was observed (AOR: 1.18, *p* = 0.001). The results showed that all multivariable logistic regression models had acceptable fits, with *p* values of the Hosmer and Lemeshow tests ranging from 0.09 to 0.99 (Table 4).

## 4. Discussion

This is one of the first studies investigating associations between self-compassion and vaccination behaviors, offering a new angle for promoting vaccination uptake among older adults. The findings also contributed to the literature on the role of self-compassion in health-related behaviors. The population-based sample and relatively high response rate were other strengths of this study.

Very few older adults in our study received an SIV in the 2025/26 flu season. The SIV coverage observed by this study (12.0%) was lower than the overall SIV coverage among older adults in China [17,18], and was much lower than the 75% coverage target recommended by the WHO [19]. The PV coverage in this study was similar to that of older adults in Shanghai (11.0%) [28], but was lower than that of older adults in Hong Kong (35.3%) [29]. About half of the participants intended to receive a fully subsidized SIV or PV. Such levels of behavioral intention were similar to those of older adults in other parts of China [17,18,23,24,25,26,27,28,29,30,31]. Previous studies showed that only half of the participants with a behavioral intention would perform the actual behavior [41]. Therefore, interventions promoting SIV and PV are needed even after fully subsidized vaccines become available. Among the participants, 49.7% intended to receive a fully subsidized RSV vaccine. Such a level of behavioral intention was slightly lower than that of older adults living in Shenzhen (60.1%) [32]. Xinjiang is less developed than Shenzhen. Therefore, it is possible that the performance of its healthcare system and health literacy of residents would be lower in Xinjiang, and hence residents may have a lower intention of receiving an RSV vaccine.

In line with previous findings, higher education level was associated with higher odds of behavioral intention to receive SIV [42]. Future SIV promotion programs should give more attention to older adults with lower education levels. As compared to older adults who were retired, those who were employed were less likely to report an intention to receive an SIV or RSV vaccine. Inflexible schedules, limited time off and inconvenience might contribute to the lower intention to receive vaccination among the working population [43]. More than 90% of the residents of Kashgar are of the Uyghur ethnic group. As compared to the Han ethnic group, people who are Uyghur were less likely to have an intention to receive an SIV. Previous studies showed that cultural and religious reasons for vaccine hesitancy were associated with lower vaccination uptake among ethnic minorities [44]. It is necessary to explore specific barriers to receiving SIV among Uyghur people in order to develop more culturally relevant health promotion programs. The majority of the participants were living with their spouses. Living with a spouse was associated with higher odds of behavioral intention to receive an SIV, PV, and RSV vaccine. Protecting a spouse might be a motivation for older adults to receive these vaccines. In addition, older adults who were living with children or grandchildren were more likely to have an intention to receive an RSV vaccine. During the study period, RSV vaccination was only available to children under the age of 12 months in China, with limited coverage. It is likely that protecting children or grandchildren from RSV infection might motivate older adults to receive an RSV vaccine. Presence of any chronic conditions was also associated with a higher intention to receive an RSV vaccine. These older adults might have believed that they were more vulnerable to severe consequences of infectious diseases due to poorer health conditions. Participants who reported a history of confirmed SARS-CoV-2 infection were less likely to report an intention to receive an RSV vaccine. Previous studies suggested that people with a prior history of infection were likely to believe that they already possessed sufficient immunity induced by natural progression and hence perceive additional vaccination as unnecessary [45]. Such a reason might also explain the negative correlation between history of PD and intention to receive a PV. In line with previous findings, a history of COVID-19 vaccination was associated with higher odds of intention to receive all three types of vaccines [24]. Older adults who received the COVID-19 vaccine primary series or booster doses might be more motivated to use vaccination to prevent infectious diseases. SIV uptake was associated with higher odds of receiving a PV. SIV and PV can be safely administered together at the same time to enhance protection, particularly among older adults [46]. Some regions are implementing the co-administration approach, which may improve uptake of both SIV and PV [47].

All six components of self-compassion were associated with a behavioral intention to receive at least one type of vaccine among our participants, which suggested a new angle for vaccination promotion targeting older adults. A higher level of self-kindness and a lower level of self-judgment were associated with higher odds of receiving all three types of vaccines. Kindness to oneself (self-kindness) or not being overly self-critical (lack of self-judgment) could facilitate effective self-regulation, leading individuals to adopt health promoting behaviors (e.g., take up vaccination) to care for their physical wellbeing [48,49]. People who are upset, frustrated, or feeling depressed are more likely to engage in self-defeating behaviors (e.g., hesitancy to receive a vaccine). Mindfulness or not overly identifying may attenuate the impact of these negative effects on effective self-regulation [49]. This may explain the significant and positive correlation between mindfulness or over-identification and behavioral intention to receive an SIV, PV or RSV vaccine. Higher self-compassion may be a part of an empathetic approach to health. People with higher common humanity or lower isolation might be more likely to receive a vaccination to protect themselves and others [48,49]. In this study, a higher level of common humanity or a lower level of isolation was associated with higher odds of receiving an SIV or PV. Self-compassion is a skill that can be learned rather than a permanent personal trait [50]. A meta-analysis suggested that various types of self-compassion interventions were effective in increasing levels of self-compassion across different populations [50]. In Kashgar, service providers in the community health centers proactively reach out to local families for health management on a regular basis. Therefore, intervention sessions led by community health center staff may be a feasible option to improve self-compassion in Kashgar. Previous studies supported the feasibility and effectiveness of community health worker-led mindful self-compassion programs [51,52]. Thus, self-compassion as a framework can be incorporated into future vaccination promotion programs in addition to the frameworks mostly adopted, such as the health belief model or the stage of change model.

This study had several limitations. First, we did not include older inpatients or residents of residential care homes. Therefore, the findings could not be generalized to all older adults living in Kashgar. Nonetheless, the distribution of sociodemographic and health characteristics of the participants was similar to that reported in a large survey of older adults in Xinjiang [53]. Second, we were not able to collect information from refusals. It is possible that refusals might have different characteristics as compared to participants. Self-selection bias existed. Third, Xinjiang is a highly diverse region inhabited by over 40 distinct ethnic groups. Therefore, caution is needed when generalizing the findings to all older adults in Xinjiang due to the differences in health literacy and cultural factors. Moreover, data were collected through face-to-face interviews. Participants may have over-reported vaccination intention due to social desirability. Finally, this was a cross-sectional survey and could not establish a causal relationship.

## 5. Conclusions

In sum, this study provides useful findings concerning the effectiveness of current SIV and PV programs for older adults in Western China. The findings also suggested that providing fully subsidized vaccines might not guarantee a high uptake rate among older adults. Health promotion is necessary even after the rollout of a fully subsidized vaccination program by the government. A higher level of self-compassion was associated with a higher intention to receive fully subsidized SIV, PV and RSV vaccines. Given the close connection and rapport established between community health centers and local families, intervention sessions led by community health center staff may be a feasible option to improve self-compassion in Kashgar. In the future, a cluster randomized controlled trial may be considered to evaluate the efficacy of self-compassion interventions in improving vaccination uptake compared to the standard of care.

## Figures and Tables

**Table 1:** Background characteristics of the participants (n = 692).

	n	%
**Socio-demographic characteristics**		
Age groups, years		
60–64	124	17.9
65–70	351	50.7
71–74	99	14.3
75 or above	118	17.1
Sex assigned at birth		
Male	333	48.1
Female	359	51.9
Education level		
Junior high or below	592	85.5
Senior high or equivalent	66	9.5
College and above	34	4.9
Marital status		
Married	542	78.3
Single, divorced, or widowed	150	21.7
Employment status		
Retired	527	76.2
Full-time/part-time/self-employed	165	23.8
Monthly personal income, CNY (US$)		
Below 3000 CNY (US$426.1)	487	70.4
3000–4999 CNY (US$426.1–710.1)	126	18.2
5000 CNY or above (US$710.2)	79	11.4
Ethnicity		
Uyghur	640	92.5
Han	49	7.1
Others	3	0.4
Living with the following people, yes		
Spouse	417	60.3
Children	292	42.2
Grandchildren	171	24.7
Other relatives	21	3.0
Domestic helpers	4	0.6
Other people	14	2.0
**Lifestyles and health conditions**		
Smoking in the past year		
No	578	83.5
Yes	114	16.5
Binge drinking in the past year		
No	656	94.8
Yes	36	5.2
Presence of chronic conditions, yes		
Hypertension	422	61.0
Other cardiovascular diseases	158	22.8
Chronic lung diseases	81	11.7
Chronic liver diseases	60	8.7
Chronic kidney diseases	91	13.2
Diabetes mellitus	131	18.9
Any of above	500	72.3
History of confirmed SARS-CoV-2 infection		
No/uncertain	250	36.1
Yes	442	63.9
History of confirmed seasonal influenza infection since September 2025		
No/uncertain	594	85.8
Yes	98	14.2
History of confirmed invasive pneumococcal diseases		
No/uncertain	673	97.3
Yes	19	2.7
History of confirmed respiratory syncytial virus infection		
No/uncertain	649	93.8
Yes	43	6.2
**Vaccination history**		
Uptake of seasonal influenza vaccination since September 2025		
No/uncertain	609	88.0
Yes	83	12.0
Uptake of pneumococcal vaccination in lifetime		
No/uncertain	587	84.8
Yes	105	15.2
Uptake of respiratory syncytial virus vaccines		
No/uncertain	692	100.0
Yes	0	0.0
Number of doses of COVID-19 vaccination received		
0–1	122	17.6
2	174	25.1
3 or above	396	57.2

**Table 2:** Behavioral intention to receive different types of vaccination and descriptive statistics of self-compassion.

	N (%)	Mean (SD)
**Behavioral intention to receive different types of vaccinations**		
Likelihood of receiving a fully subsidized seasonal influenza vaccination provided by the government in the next year		
Very unlikely/unlikely/neutral	301 (43.5)	N.A.
Likely/very likely	391 (56.5)	N.A.
Likelihood of receiving a fully subsidized pneumococcal vaccination provided by the government in the next year (among 587 participants who have never received such a vaccine)		
Very unlikely/unlikely/neutral	304 (51.8)	N.A.
Likely/very likely	283 (48.2)	N.A.
Likelihood of receiving a fully subsidized respiratory syncytial virus vaccination provided by the government in the next year (among 692 participants who have never received such a vaccine)		
Very unlikely/unlikely/neutral	348 (50.3)	N.A.
Likely/very likely	344 (49.7)	N.A.
**Self-compassion Scale Short Form ^1^**		
Self-kindness Items	N.A.	4.8 (1.6)
Self-judgment Items	N.A.	4.6 (1.7)
Common Humanity Items	N.A.	4.7 (1.7)
Isolation Items	N.A.	4.8 (1.6)
Mindfulness Items	N.A.	5.0 (1.7)
Over-identification Items	N.A.	4.6 (1.5)

N.A.: not applicable. ^1^. Score of each component of the Self-compassion Scale Short Form was calculated by summing up two individual item scores. The possible range of scores of each component was between 2 and 10. Higher scores in self-kindness, common humanity, and mindfulness represented higher levels of self-kindness, common humanity, and mindfulness. Higher scores in self-judgment, isolation and over-identification indicated lower levels of self-judgment, isolation and over-identification.

**Table 3:** Associations between background characteristics and behavioral intention to receive different types of vaccination.

	Behavioral Intention to Receive a Fully Subsidized Seasonal Influenza Vaccination Provided by the Government in the Next Year (Among All 692 Participants)		Behavioral Intention to Receive a Fully Subsidized Pneumococcal Vaccination Provided by the Government in the Next year (Among 587 Participants Who Have Never Received Such Vaccine)		Behavioral Intention to Receive a Fully Subsidized Respiratory Syncytial Virus Vaccination Provided by the Government in the Next Year (Among 692 Participants Who Have Never Received Such Vaccine)	
	OR (95% CI)	*p* Values	OR (95% CI)	*p* Values	OR (95% CI)	*p* Values
**Socio-demographic characteristics**						
Age groups, years						
60–64	Reference		Reference		Reference	
65–70	0.91 (0.59, 1.40)	0.67	0.93 (0.61, 1.42)	0.73	1.01 (0.66, 1.53)	0.97
71–74	0.74 (0.43, 1.28)	0.74	1.02 (0.60, 1.75)	0.94	0.68 (0.42, 1.11)	0.12
75 or above	1.05 (0.62, 1.78)	0.85	0.84 (0.50, 1.41)	0.52	1.00 (0.61, 1.66)	0.99
Sex assigned at birth						
Male	Reference		Reference		Reference	
Female	1.05 (0.78, 1.42)	0.74	1.11 (0.81, 1.54)	0.51	1.14 (0.84, 1.53)	0.40
Education level						
Junior high or below	Reference		Reference		Reference	
Senior high or equivalent	1.45 (0.86, 2.45)	0.17	1.07 (0.61, 1.90)	0.81	1.01 (0.61, 1.67)	0.98
College and above	2.30 (1.05, 5.01)	0.04	0.80 (0.33, 1.94)	0.63	0.90 (0.45, 1.79)	0.75
Employment status						
Retired	Reference		Reference		Reference	
Full-time/part-time/self-employed	0.47 (0.33, 0.67)	<0.001	0.93 (0.64, 1.36)	0.71	0.64 (0.45, 0.91)	0.01
Monthly personal income, CNY (US$)						
Below 3000 CNY (US$426.1)	Reference		Reference		Reference	
3000–4999 CNY (US$426.1–710.1)	1.34 (0.90, 2.00)	0.15	1.06 (0.69, 1.63)	0.78	1.17 (0.79, 1.73)	0.44
5000 CNY or above (US$710.2)	1.15 (0.71, 1.86)	0.57	1.18 (0.69, 2.03)	0.55	0.91 (0.56, 1.46)	0.69
Ethnicity						
Uyghur	Reference		Reference		Reference	
Han	2.83 (1.42, 5.63)	0.003	1.01 (0.50, 2.04)	0.97	1.06 (0.59, 1.90)	0.84
Others	0.41 (0.04, 3.53)	0.47	1.08 (0.07, 12.28)	0.96	2.04 (0.18, 22.59)	0.56
Living with the following people, yes						
Spouse	1.69 (1.24, 2.30)	<0.001	1.48 (1.06, 2.06)	0.02	2.02 (1.48, 2.75)	<0.001
Children	1.10 (0.81, 1.49)	0.53	1.25 (0.90, 1.73)	0.18	1.68 (1.24, 2.28)	<0.001
Grandchildren	1.27 (0.89, 1.80)	0.19	1.21 (0.83, 1.75)	0.33	1.76 (1.24, 2.50)	0.002
**Lifestyles and health conditions**						
Presence of any chronic conditions						
No	Reference		Reference		Reference	
Yes	1.11 (0.80, 1.55)	0.54	1.04 (0.73, 1.49)	0.81	1.71 (1.22, 2.39)	0.002
History of confirmed SARS-CoV-2 infection						
No	Reference		Reference		Reference	
Yes	1.01 (0.74, 1.38)	0.97	0.89 (0.64, 1.25)	0.51	0.69 (0.51, 0.94)	0.02
History of confirmed seasonal influenza infection since September 2025						
No/uncertain	Reference		Reference		Reference	
Yes	1.90 (1.20, 3.01)	0.01	1.19 (0.73, 1.95)	0.48	1.49 (0.97, 2.29)	0.07
History of confirmed invasive pneumococcal diseases						
No/uncertain	Reference		Reference		Reference	
Yes	1.33 (0.52, 3.42)	0.55	0.19 (0.04, 0.86)	0.03	1.40 (0.56, 3.53)	0.47
**Vaccination history**						
Uptake of seasonal influenza vaccination since September 2025						
No/uncertain	Reference		Reference		Reference	
Yes	1.81 (1.11, 2.95)	0.02	4.01 (2.06, 7.81)	<0.001	1.45 (0.91, 2.31)	0.12
Number of doses of COVID-19 vaccination received						
0–1	Reference		Reference		Reference	
2	2.70 (1.67, 4.36)	<0.001	3.09 (1.80, 5.29)	<0.001	2.18 (1.36, 3.51)	0.001
3 or above	3.16 (2.06, 4.83)	<0.001	2.78 (1.72, 4.49)	<0.001	1.88 (1.24, 2.86)	0.003

OR: crude odds ratios; CI: confidence interval.

**Table 4:** Associations between self-compassion and behavioral intention to receive different types of vaccination.

	Behavioral Intention to Receive a Fully Subsidized Seasonal Influenza Vaccination Provided by the Government in the Next Year (Among All 692 Participants)			Behavioral Intention to Receive a Fully Subsidized Pneumococcal Vaccination Provided by the Government in the Next Year (Among 587 Participants Who Have Never Received Such a Vaccine)			Behavioral Intention to Receive a Fully Subsidized Respiratory Syncytial Virus Vaccination Provided by the Government in the Next Year (Among 692 Participants Who Have Never Received Such a Vaccine)		
	AOR (95% CI)	*p* Values	Hosmer & Lemeshow Test	AOR (95% CI)	*p* Values	Hosmer & Lemeshow Test	AOR (95% CI)	*p* Values	Hosmer & Lemeshow Test
Self-kindness Items	1.28 (1.14, 1.42)	<0.001	0.73	1.22 (1.09, 1.37)	<0.001	0.99	1.03 (0.93, 1.13)	0.61	0.13
Self-judgment Items	1.14 (1.03, 1.26)	0.01	0.65	1.09 (0.98, 1.21)	0.12	0.17	1.17 (1.06, 1.28)	0.002	0.19
Common Humanity Items	1.18 (1.07, 1.30)	0.001	0.17	1.11 (1.00, 1.23)	0.053	0.16	1.04 (0.94, 1.14)	0.47	0.13
Isolation Items	1.19 (1.07, 1.32)	0.001	0.31	1.25 (1.11, 1.39)	<0.001	0.72	1.09 (0.99, 1.20)	0.08	0.14
Mindfulness Items	1.26 (1.14, 1.39)	<0.001	0.75	1.21 (1.09, 1.34)	<0.001	0.15	1.15 (1.05, 1.26)	0.004	0.46
Over-identification Items	1.30 (1.16, 1.47)	<0.001	0.78	1.18 (1.04, 1.33)	0.01	0.27	1.23 (1.10, 1.38)	<0.001	0.09

AOR: adjusted odds ratios, adjusted for significant background characteristics listed in Table 3. CI: confidence interval. Higher scores in self-kindness, common humanity, and mindfulness represented higher levels of self-kindness, common humanity, and mindfulness. Higher scores in self-judgment, isolation and over-identification indicated a lower level of self-judgment, isolation and over-identification.

## Data Availability

The datasets generated and/or analyzed during the current study are not publicly available as they contain sensitive personal information but are available from the corresponding author upon reasonable request.

## Abbreviations

The following abbreviations are used in this manuscript:

AOR	Adjusted odds ratio
CDC	Centers for Disease Control and Prevention
CI	Confidence interval
CNY	Chinese Yuan
IPD	Invasive pneumococcal diseases
OR	Crude odds ratio
PD	Pneumococcal diseases
PV	Pneumococcal vaccination
RSV	Respiratory syncytial virus
SCS-SF	Self-Compassion Scale–Short Form
SD	Standard deviation
SIV	Seasonal influenza vaccination
US$	United States dollar
WHO	World Health Organization

## Appendix A

**Table A1:** Associations between all background characteristics and behavioral intention to receive different types of vaccination.

	Behavioral Intention to Receive a Fully Subsidized Seasonal Influenza Vaccination Provided by the Government in the Next Year (Among All 692 Participants)		Behavioral Intention to Receive a Fully Subsidized Pneumococcal Vaccination Provided by the Government in the Next Year (Among 587 Participants Who Have Never Received Such Vaccine)		Behavioral Intention to Receive a Fully Subsidized Respiratory Syncytial Virus Vaccination Provided by the Government in the Next Year (Among 692 Participants Who Have Never Received Such Vaccine)	
	OR (95% CI)	*p* Values	OR (95% CI)	*p* Values	OR (95% CI)	*p* Values
**Socio-demographic characteristics**						
Age groups, years						
60–64	Reference		Reference		Reference	
65–70	0.91 (0.59, 1.40)	0.67	0.93 (0.61, 1.42)	0.73	1.01 (0.66, 1.53)	0.97
71–74	0.74 (0.43, 1.28)	0.74	1.02 (0.60, 1.75)	0.94	0.68 (0.42, 1.11)	0.12
75 or above	1.05 (0.62, 1.78)	0.85	0.84 (0.50, 1.41)	0.52	1.00 (0.61, 1.66)	0.99
Sex assigned at birth						
Male	Reference		Reference		Reference	
Female	1.05 (0.78, 1.42)	0.74	1.11 (0.81, 1.54)	0.51	1.14 (0.84, 1.53)	0.40
Education level						
Junior high or below	Reference		Reference		Reference	
Senior high or equivalent	1.45 (0.86, 2.45)	0.17	1.07 (0.61, 1.90)	0.81	1.01 (0.61, 1.67)	0.98
College and above	2.30 (1.05, 5.01)	0.04	0.80 (0.33, 1.94)	0.63	0.90 (0.45, 1.79)	0.75
Marital status						
Married	Reference		Reference		Reference	
Single, divorced, or widowed	1.01 (0.70, 1.45)	0.96	0.89 (0.61, 1.31)	0.55	0.80 (0.56, 1.15)	0.23
Employment status						
Retired	Reference		Reference		Reference	
Full-time/part-time/self-employed	0.47 (0.33, 0.67)	<0.001	0.93 (0.64, 1.36)	0.71	0.64 (0.45, 0.91)	0.01
Monthly personal income, CNY (US$)						
Below 3000 CNY (US$426.1)	Reference		Reference		Reference	
3000–4999 CNY (US$426.1–710.1)	1.34 (0.90, 2.00)	0.15	1.06 (0.69, 1.63)	0.78	1.17 (0.79, 1.73)	0.44
5000 CNY or above (US$710.2)	1.15 (0.71, 1.86)	0.57	1.18 (0.69, 2.03)	0.55	0.91 (0.56, 1.46)	0.69
Ethnicity						
Uyghur	Reference		Reference		Reference	
Han	2.83 (1.42, 5.63)	0.003	1.01 (0.50, 2.04)	0.97	1.06 (0.59, 1.90)	0.84
Others	0.41 (0.04, 3.53)	0.47	1.08 (0.07, 12.28)	0.96	2.04 (0.18, 22.59)	0.56
Living with the following people, yes						
Spouse	1.69 (1.24, 2.30)	<0.001	1.48 (1.06, 2.06)	0.02	2.02 (1.48, 2.75)	<0.001
Children	1.10 (0.81, 1.49)	0.53	1.25 (0.90, 1.73)	0.18	1.68 (1.24, 2.28)	<0.001
Grandchildren	1.27 (0.89, 1.80)	0.19	1.21 (0.83, 1.75)	0.33	1.76 (1.24, 2.50)	0.002
Other relatives	2.53 (0.92, 6.98)	0.07	0.37 (0.13, 1.05)	0.06	0.75 (0.31, 1.81)	0.53
Domestic helpers	N.A.	N.A.	2.16 (0.19, 23.91)	0.53	1.01 (0.14, 7.22)	0.99
Other people	1.95 (0.61, 6.28)	0.26	2.17 (0.54, 8.77)	0.28	1.36 (0.47, 3.95)	0.58
**Lifestyles and health conditions**						
Smoking in the past year						
No	Reference		Reference		Reference	
Yes	0.94 (0.63, 1.41)	0.77	1.04 (0.67, 1.60)	0.87	1.15 (0.77, 1.72)	0.50
Binge drinking in the past year						
No	Reference		Reference		Reference	
Yes	0.85 (0.44, 1.67)	0.64	1.25 (0.59, 2.68)	0.56	1.28 (0.65, 2.52)	0.47
Presence of any chronic conditions						
No	Reference		Reference		Reference	
Yes	1.11 (0.80, 1.55)	0.54	1.04 (0.73, 1.49)	0.81	1.71 (1.22, 2.39)	0.002
History of confirmed SARS-CoV-2 infection						
No	Reference		Reference		Reference	
Yes	1.01 (0.74, 1.38)	0.97	0.89 (0.64, 1.25)	0.51	0.69 (0.51, 0.94)	0.02
History of confirmed seasonal influenza infection since September 2025						
No/uncertain	Reference		Reference		Reference	
Yes	1.90 (1.20, 3.01)	0.01	1.19 (0.73, 1.95)	0.48	1.49 (0.97, 2.29)	0.07
History of confirmed invasive pneumococcal diseases						
No/uncertain	Reference		Reference		Reference	
Yes	1.33 (0.52, 3.42)	0.55	0.19 (0.04, 0.86)	0.03	1.40 (0.56, 3.53)	0.47
History of confirmed respiratory syncytial virus infection						
No/uncertain	Reference		Reference		Reference	
Yes	0.80 (0.43, 1.47)	0.47	1.72 (0.66, 4.49)	0.27	1.44 (0.77, 2.69)	0.26
**Vaccination history**						
Uptake of seasonal influenza vaccination since September 2025						
No/uncertain	Reference		Reference		Reference	
Yes	1.81 (1.11, 2.95)	0.02	4.01 (2.06, 7.81)	<0.001	1.45 (0.91, 2.31)	0.12
Uptake of pneumococcal vaccination in lifetime						
No/uncertain	Reference		N.A.		Reference	
Yes	0.79 (0.52, 1.19)	0.26	N.A.	N.A.	1.42 (0.94, 2.16)	0.10
Uptake of respiratory syncytial virus vaccines						
No/uncertain	Reference		Reference		N.A.	
Yes	N.A.	N.A.	N.A.	N.A.	N.A.	N.A.
Number of doses of COVID-19 vaccination received						
0–1	Reference		Reference		Reference	
2	2.70 (1.67, 4.36)	<0.001	3.09 (1.80, 5.29)	<0.001	2.18 (1.36, 3.51)	0.001
3 or above	3.16 (2.06, 4.83)	<0.001	2.78 (1.72, 4.49)	<0.001	1.88 (1.24, 2.86)	0.003

OR: crude odds ratios; CI: confidence interval; N.A.: not applicable.
